# Supplementary material for: Reporting and methodological quality of meta-analyses in urological literature
Source: PeerJ. 2017 Apr 19;5:e3129. doi: 10.7717/peerj.3129 (PMC5399869; doi:10.7717/peerj.3129)
Supplement: Supplemental Information 1 [file peerj-05-3129-s001.docx]

**Supplemental Information**

**PRISMA checklist**

1. **Title:** Identify the report as a systematic review, meta-analysis, or both.
2. **Structured summary:** Provide a structured summary including, as applicable: background; objectives; data sources; study eligibility criteria, participants, and interventions; study appraisal and synthesis methods; results; limitations; conclusions and implications of key findings; systematic review registration number
3. **Rationale:** Describe the rationale for the review in the context of what is already known.
4. **Objectives**: Provide an explicit statement of questions being addressed with reference to participants, interventions, comparisons, outcomes, and study design (PICOS).
5. **Protocol and registration:** Indicate if a review protocol exists, if and where it can be accessed (e.g., Web address), and, if available, provide registration information including registration number.
6. **Eligibility criteria:** Specify study characteristics (e.g., PICOS, length of follow-up) and report characteristics (e.g., years considered, language, publication status) used as criteria for eligibility, giving rationale.
7. **Information sources:** Describe all information sources (e.g., databases with dates of coverage, contact with study authors to identify additional studies) in the search and date last searched.
8. **Search:** Present full electronic search strategy for at least one database, including any limits used, such that it could be repeated.
9. **Study selection:** State the process for selecting studies (i.e., screening, eligibility, included in systematic review, and, if applicable, included in the meta-analysis).
10. **Data collection process:** Describe method of data extraction from reports (e.g., piloted forms, independently, in duplicate) and any processes for obtaining and confirming data from investigators.
11. **Data items:** List and define all variables for which data were sought (e.g., PICOS, funding sources) and any assumptions and simplifications made.
12. **Risk of bias in individual studies:** Describe methods used for assessing risk of bias of individual studies (including specification of whether this was done at the study or outcome level), and how this information is to be used in any data synthesis.
13. **Summary measures:** State the principal summary measures (e.g., risk ratio, difference in means).
14. **Synthesis of results:** Describe the methods of handling data and combining results of studies, if done, including measures of consistency (e.g., I^2^) for each meta-analysis.
15. **Risk of bias across studies:** Specify any assessment of risk of bias that may affect the cumulative evidence (e.g., publication bias, selective reporting within studies).
16. **Additional analyses:** Describe methods of additional analyses (e.g., sensitivity or subgroup analyses, meta-regression), if done, indicating which were pre-specified.
17. **Study selection:** Give numbers of studies screened, assessed for eligibility, and included in the review, with reasons for exclusions at each stage, ideally with a flow diagram.
18. **Study characteristics:** For each study, present characteristics for which data were extracted (e.g., study size, PICOS, follow-up period) and provide the citations.
19. **Risk of bias within studies:** Present data on risk of bias of each study and, if available, any outcome level assessment (see item 12).
20. **Results of individual studies:** For all outcomes considered (benefits or harms), present, for each study: (a) simple summary data for each intervention group (b) effect estimates and confidence intervals, ideally with a forest plot.
21. **Synthesis of results:** Present results of each meta-analysis done, including confidence intervals and measures of consistency.
22. **Risk of bias across studies:** Present results of any assessment of risk of bias across studies (see Item 15).
23. **Additional analysis:** Give results of additional analyses, if done (e.g., sensitivity or subgroup analyses, meta-regression [see Item 16]).
24. **Summary of evidence:** Summarize the main findings including the strength of evidence for each main outcome; consider their relevance to key groups (e.g., healthcare providers, users, and policy makers).
25. **Limitations:** Discuss limitations at study and outcome level (e.g., risk of bias), and at review-level (e.g., incomplete retrieval of identified research, reporting bias).
26. **Conclusions:** Provide a general interpretation of the results in the context of other evidence, and implications for future research.
27. **Funding:** Describe sources of funding for the systematic review and other support (e.g., supply of data); role of funders for the systematic review.

From: Moher, D, Liberati, A, Tetzlaff, J et al: Preferred reporting items for systematic reviews and

meta-analyses: the PRISMA statement. PLoS Med 2009, **6:** e1000097

**AMSTAR tool**

1. **Was an 'a priori' design provided?**

The research question and inclusion criteria should be established before the conduct of the review.

1. **Was there duplicate study selection and data extraction?**

There should be at least two independent data extractors and a consensus procedure for disagreements should be in place.

1. **Was a comprehensive literature search performed?**

At least two electronic sources should be searched. The report must include years and databases used (e.g. Central, EMBASE, and MEDLINE). Key words and/or MESH terms must be stated and where feasible the search strategy should be provided. All searches should be supplemented by consulting current contents, reviews, textbooks, specialized registers, or experts in the particular field of study, and by reviewing the references in the studies found.

1. **Was the status of publication (i.e. grey literature) used as an inclusion criterion?**

The authors should state that they searched for reports regardless of their publication type. The authors should state whether or not they excluded any reports (from the systematic review), based on their publication status, language etc.

1. **Was a list of studies (included and excluded) provided?**

A list of included and excluded studies should be provided.

1. **Were the characteristics of the included studies provided?**

In an aggregated form such as a table, data from the original studies should be provided on the participants, interventions and outcomes. The ranges of characteristics in all the studies analyzed e.g. age, race, sex, relevant socioeconomic data, disease status, duration, severity, or other diseases should be reported.

1. **Was the scientific quality of the included studies assessed and documented?**

'A priori' methods of assessment should be provided (e.g., for effectiveness studies if the author(s) chose to include only randomized, double-blind, placebo controlled studies, or allocation concealment as inclusion criteria); for other types of studies alternative items will be relevant.

1. **Was the scientific quality of the included studies used appropriately in formulating conclusions?**

The results of the methodological rigor and scientific quality should be considered in the analysis and the conclusions of the review, and explicitly stated in formulating recommendations.

1. **Were the methods used to combine the findings of studies appropriate?**

For the pooled results, a test should be done to ensure the studies were combinable, to assess their homogeneity (i.e. Chi-squared test for homogeneity, I^2^). If heterogeneity exists a random effects model should be used and/or the clinical appropriateness of combining should be taken into consideration (i.e. is it sensible to combine?)

1. **Was the likelihood of publication bias assessed?**

An assessment of publication bias should include a combination of graphical aids (e.g., funnel plot, other available tests) and/or statistical tests (e.g., Egger regression test).

1. **Was the conflict of interest stated?**

Potential sources of support should be clearly acknowledged in both the systematic review and the included studies.

Each items is given four responses**: Yes, No, Can’t answer, Not applicable**

From: Shea, BJ, Grimshaw, JM, Wells, GA et al: Development of AMSTAR: a measurement tool to assess the methodological quality of systematic reviews. BMC Med Res Methodol 2007, **7:** 10

**Included meta-analyses ^1-183^**

1. Gacci, M, Corona, G, Vignozzi, L et al: Metabolic syndrome and benign prostatic enlargement: a systematic review and meta-analysis. BJU Int 2015, **115:** 24

2. Bates, AS, Martin, RM, Terry, TR: Complications following artificial urinary sphincter placement after radical prostatectomy and radiotherapy: a meta-analysis. BJU Int 2015, **116:** 623

3. Autorino, R, Brandao, LF, Sankari, B et al: Laparoendoscopic single-site (LESS) vs laparoscopic living-donor nephrectomy: a systematic review and meta-analysis. BJU Int 2015, **115:** 206

4. van Die, MD, Bone, KM, Williams, SG et al: Soy and soy isoflavones in prostate cancer: a systematic review and meta-analysis of randomized controlled trials. BJU Int 2014, **113:** E119

5. Omar, MI, Lam, TB, Alexander, CE et al: Systematic review and meta-analysis of the clinical effectiveness of bipolar compared with monopolar transurethral resection of the prostate (TURP). BJU Int 2014, **113:** 24

6. Cundy, TP, Harling, L, Hughes-Hallett, A et al: Meta-analysis of robot-assisted vs conventional laparoscopic and open pyeloplasty in children. BJU Int 2014, **114:** 582

7. Bi, L, Huang, H, Fan, X et al: Extended vs non-extended pelvic lymph node dissection and their influence on recurrence-free survival in patients undergoing radical cystectomy for bladder cancer: a systematic review and meta-analysis of comparative studies. BJU Int 2014, **113:** E39

8. Zhang, X, Shen, Z, Zhong, S et al: Comparison of peri-operative outcomes of robot-assisted vs laparoscopic partial nephrectomy: a meta-analysis. BJU Int 2013, **112:** 1133

9. Teng, J, Zhang, D, Li, Y et al: Photoselective vaporization with the green light laser vs transurethral resection of the prostate for treating benign prostate hyperplasia: a systematic review and meta-analysis. BJU Int 2013, **111:** 312

10. Scosyrev, E, Tobis, S, Donsky, H et al: Statin use and the risk of biochemical recurrence of prostate cancer after definitive local therapy: a meta-analysis of eight cohort studies. BJU Int 2013, **111:** E71

11. Robertson, C, Close, A, Fraser, C et al: Relative effectiveness of robot-assisted and standard laparoscopic prostatectomy as alternatives to open radical prostatectomy for treatment of localised prostate cancer: a systematic review and mixed treatment comparison meta-analysis. BJU Int 2013, **112:** 798

12. Mohsen, B, Giorgio, T, Rasoul, ZS et al: Application of C-11-acetate positron-emission tomography (PET) imaging in prostate cancer: systematic review and meta-analysis of the literature. BJU Int 2013, **112:** 1062

13. Meurs, P, Galvin, R, Fanning, DM et al: Prognostic value of the CAPRA clinical prediction rule: a systematic review and meta-analysis. BJU Int 2013, **111:** 427

14. Kahokehr, A, Vather, R, Nixon, A et al: Non-steroidal anti-inflammatory drugs for lower urinary tract symptoms in benign prostatic hyperplasia: systematic review and meta-analysis of randomized controlled trials. BJU Int 2013, **111:** 304

15. Houghton, BB, Chalasani, V, Hayne, D et al: Intravesical chemotherapy plus bacille Calmette-Guerin in non-muscle invasive bladder cancer: a systematic review with meta-analysis. BJU Int 2013, **111:** 977

16. Froghi, S, Ahmed, K, Khan, MS et al: Evaluation of robotic and laparoscopic partial nephrectomy for small renal tumours (T1a). BJU Int 2013, **112:** E322

17. Fan, X, Xu, K, Lin, T et al: Comparison of transperitoneal and retroperitoneal laparoscopic nephrectomy for renal cell carcinoma: a systematic review and meta-analysis. BJU Int 2013, **111:** 611

18. Zheng, C, Lv, Y, Zhong, Q et al: Narrow band imaging diagnosis of bladder cancer: systematic review and meta-analysis. BJU Int 2012, **110:** E680

19. Wang, J, Zhao, C, Zhang, C et al: Tubeless vs standard percutaneous nephrolithotomy: a meta-analysis. BJU Int 2012, **109:** 918

20. Teng, J, Chen, M, Gao, Y et al: Transrectal sonoelastography in the detection of prostate cancers: a meta-analysis. BJU Int 2012, **110:** E614

21. Shen, P, Yang, J, Wei, W et al: Effects of fluorescent light-guided transurethral resection on non-muscle-invasive bladder cancer: a systematic review and meta-analysis. BJU Int 2012, **110:** E209

22. Ku, JH, Godoy, G, Amiel, GE et al: Urine survivin as a diagnostic biomarker for bladder cancer: a systematic review. BJU Int 2012, **110:** 630

23. El Dib, R, Touma, NJ, Kapoor, A: Cryoablation vs radiofrequency ablation for the treatment of renal cell carcinoma: a meta-analysis of case series studies. BJU Int 2012, **110:** 510

24. Ding, H, Tian, J, Du, W et al: Open non-microsurgical, laparoscopic or open microsurgical varicocelectomy for male infertility: a meta-analysis of randomized controlled trials. BJU Int 2012, **110:** 1536

25. Aboumarzouk, OM, Stein, RJ, Haber, GP et al: Laparoscopic partial nephrectomy in obese patients: a systematic review and meta-analysis. BJU Int 2012, **110:** 1244

26. Aboumarzouk, OM, Ogston, S, Huang, Z et al: Diagnostic accuracy of transrectal elastosonography (TRES) imaging for the diagnosis of prostate cancer: a systematic review and meta-analysis. BJU Int 2012, **110:** 1414

27. Lamb, AD, Vowler, SL, Johnston, R et al: Meta-analysis showing the beneficial effect of alpha-blockers on ureteric stent discomfort. BJU Int 2011, **108:** 1894

28. Yuh, B, Wilson, T, Bochner, B et al: Systematic review and cumulative analysis of oncologic and functional outcomes after robot-assisted radical cystectomy. Eur Urol 2015, **67:** 402

29. Vera-Badillo, FE, Templeton, AJ, Duran, I et al: Systemic therapy for non-clear cell renal cell carcinomas: a systematic review and meta-analysis. Eur Urol 2015, **67:** 740

30. Simpkin, AJ, Tilling, K, Martin, RM et al: Systematic Review and Meta-analysis of Factors Determining Change to Radical Treatment in Active Surveillance for Localized Prostate Cancer. Eur Urol 2015, **67:** 993

31. Seisen, T, Granger, B, Colin, P et al: A Systematic Review and Meta-analysis of Clinicopathologic Factors Linked to Intravesical Recurrence After Radical Nephroureterectomy to Treat Upper Tract Urothelial Carcinoma. Eur Urol 2015, **67:** 1122

32. Schoots, IG, Roobol, MJ, Nieboer, D et al: Magnetic resonance imaging-targeted biopsy may enhance the diagnostic accuracy of significant prostate cancer detection compared to standard transrectal ultrasound-guided biopsy: a systematic review and meta-analysis. Eur Urol 2015, **68:** 438

33. Reeves, F, Preece, P, Kapoor, J et al: Preservation of the neurovascular bundles is associated with improved time to continence after radical prostatectomy but not long-term continence rates: results of a systematic review and meta-analysis. Eur Urol 2015, **68:** 692

34. Perera, M, Roberts, MJ, Doi, SA et al: Prostatic urethral lift improves urinary symptoms and flow while preserving sexual function for men with benign prostatic hyperplasia: a systematic review and meta-analysis. Eur Urol 2015, **67:** 704

35. Novara, G, Catto, JW, Wilson, T et al: Systematic review and cumulative analysis of perioperative outcomes and complications after robot-assisted radical cystectomy. Eur Urol 2015, **67:** 376

36. Hamoen, EH, de Rooij, M, Witjes, JA et al: Use of the Prostate Imaging Reporting and Data System (PI-RADS) for Prostate Cancer Detection with Multiparametric Magnetic Resonance Imaging: A Diagnostic Meta-analysis. Eur Urol 2015, **67:** 1112

37. De, S, Autorino, R, Kim, FJ et al: Percutaneous nephrolithotomy versus retrograde intrarenal surgery: a systematic review and meta-analysis. Eur Urol 2015, **67:** 125

38. Cornu, JN, Ahyai, S, Bachmann, A et al: A Systematic Review and Meta-analysis of Functional Outcomes and Complications Following Transurethral Procedures for Lower Urinary Tract Symptoms Resulting from Benign Prostatic Obstruction: An Update. Eur Urol 2015, **67:** 1066

39. Choi, JE, You, JH, Kim, DK et al: Comparison of perioperative outcomes between robotic and laparoscopic partial nephrectomy: a systematic review and meta-analysis. Eur Urol 2015, **67:** 891

40. Bosco, C, Bosnyak, Z, Malmberg, A et al: Quantifying observational evidence for risk of fatal and nonfatal cardiovascular disease following androgen deprivation therapy for prostate cancer: a meta-analysis. Eur Urol 2015, **68:** 386

41. Serati, M, Bogani, G, Sorice, P et al: Robot-assisted sacrocolpopexy for pelvic organ prolapse: a systematic review and meta-analysis of comparative studies. Eur Urol 2014, **66:** 303

42. Petrelli, F, Coinu, A, Cabiddu, M et al: Correlation of pathologic complete response with survival after neoadjuvant chemotherapy in bladder cancer treated with cystectomy: a meta-analysis. Eur Urol 2014, **65:** 350

43. Mostafa, A, Lim, CP, Hopper, L et al: Single-incision mini-slings versus standard midurethral slings in surgical management of female stress urinary incontinence: an updated systematic review and meta-analysis of effectiveness and complications. Eur Urol 2014, **65:** 402

44. Leow, JJ, Martin-Doyle, W, Rajagopal, PS et al: Adjuvant chemotherapy for invasive bladder cancer: a 2013 updated systematic review and meta-analysis of randomized trials. Eur Urol 2014, **66:** 42

45. Leow, JJ, Martin-Doyle, W, Fay, AP et al: A systematic review and meta-analysis of adjuvant and neoadjuvant chemotherapy for upper tract urothelial carcinoma. Eur Urol 2014, **66:** 529

46. Islami, F, Moreira, DM, Boffetta, P et al: A systematic review and meta-analysis of tobacco use and prostate cancer mortality and incidence in prospective cohort studies. Eur Urol 2014, **66:** 1054

47. Cartwright, R, Mangera, A, Tikkinen, KA et al: Systematic review and meta-analysis of candidate gene association studies of lower urinary tract symptoms in men. Eur Urol 2014, **66:** 752

48. Brandao, LF, Autorino, R, Laydner, H et al: Robotic versus laparoscopic adrenalectomy: a systematic review and meta-analysis. Eur Urol 2014, **65:** 1154

49. Autorino, R, Eden, C, El-Ghoneimi, A et al: Robot-assisted and laparoscopic repair of ureteropelvic junction obstruction: a systematic review and meta-analysis. Eur Urol 2014, **65:** 430

50. Perlis, N, Zlotta, AR, Beyene, J et al: Immediate post-transurethral resection of bladder tumor intravesical chemotherapy prevents non-muscle-invasive bladder cancer recurrences: an updated meta-analysis on 2548 patients and quality-of-evidence review. Eur Urol 2013, **64:** 421

51. Jiang, X, Zhu, S, Feng, G et al: Is an initial saturation prostate biopsy scheme better than an extended scheme for detection of prostate cancer? A systematic review and meta-analysis. Eur Urol 2013, **63:** 1031

52. Evangelista, L, Guttilla, A, Zattoni, F et al: Utility of choline positron emission tomography/computed tomography for lymph node involvement identification in intermediate- to high-risk prostate cancer: a systematic literature review and meta-analysis. Eur Urol 2013, **63:** 1040

53. Cui, Y, Zhang, Y: The effect of androgen-replacement therapy on prostate growth: a systematic review and meta-analysis. Eur Urol 2013, **64:** 811

54. Burger, M, Grossman, HB, Droller, M et al: Photodynamic diagnosis of non-muscle-invasive bladder cancer with hexaminolevulinate cystoscopy: a meta-analysis of detection and recurrence based on raw data. Eur Urol 2013, **64:** 846

55. Agur, W, Riad, M, Secco, S et al: Surgical treatment of recurrent stress urinary incontinence in women: a systematic review and meta-analysis of randomised controlled trials. Eur Urol 2013, **64:** 323

56. Thangasamy, IA, Chalasani, V, Bachmann, A et al: Photoselective vaporisation of the prostate using 80-W and 120-W laser versus transurethral resection of the prostate for benign prostatic hyperplasia: a systematic review with meta-analysis from 2002 to 2012. Eur Urol 2012, **62:** 315

57. Tewari, A, Sooriakumaran, P, Bloch, DA et al: Positive surgical margin and perioperative complication rates of primary surgical treatments for prostate cancer: a systematic review and meta-analysis comparing retropubic, laparoscopic, and robotic prostatectomy. Eur Urol 2012, **62:** 1

58. Rocco, B, Cozzi, G, Spinelli, MG et al: Posterior musculofascial reconstruction after radical prostatectomy: a systematic review of the literature. Eur Urol 2012, **62:** 779

59. Novara, G, Ficarra, V, Rosen, RC et al: Systematic review and meta-analysis of perioperative outcomes and complications after robot-assisted radical prostatectomy. Eur Urol 2012, **62:** 431

60. Novara, G, Ficarra, V, Mocellin, S et al: Systematic review and meta-analysis of studies reporting oncologic outcome after robot-assisted radical prostatectomy. Eur Urol 2012, **62:** 382

61. Ni, S, Tao, W, Chen, Q et al: Laparoscopic versus open nephroureterectomy for the treatment of upper urinary tract urothelial carcinoma: a systematic review and cumulative analysis of comparative studies. Eur Urol 2012, **61:** 1142

62. Madhuvrata, P, Singh, M, Hasafa, Z et al: Anticholinergic drugs for adult neurogenic detrusor overactivity: a systematic review and meta-analysis. Eur Urol 2012, **62:** 816

63. Gacci, M, Corona, G, Salvi, M et al: A systematic review and meta-analysis on the use of phosphodiesterase 5 inhibitors alone or in combination with alpha-blockers for lower urinary tract symptoms due to benign prostatic hyperplasia. Eur Urol 2012, **61:** 994

64. Ficarra, V, Novara, G, Rosen, RC et al: Systematic review and meta-analysis of studies reporting urinary continence recovery after robot-assisted radical prostatectomy. Eur Urol 2012, **62:** 405

65. Ficarra, V, Novara, G, Ahlering, TE et al: Systematic review and meta-analysis of studies reporting potency rates after robot-assisted radical prostatectomy. Eur Urol 2012, **62:** 418

66. Fan, X, Lin, T, Xu, K et al: Laparoendoscopic single-site nephrectomy compared with conventional laparoscopic nephrectomy: a systematic review and meta-analysis of comparative studies. Eur Urol 2012, **62:** 601

67. Aboumarzouk, OM, Stein, RJ, Eyraud, R et al: Robotic versus laparoscopic partial nephrectomy: a systematic review and meta-analysis. Eur Urol 2012, **62:** 1023

68. Liu, Y, Hu, F, Li, D et al: Does physical activity reduce the risk of prostate cancer? A systematic review and meta-analysis. Eur Urol 2011, **60:** 1029

69. Goossens-Laan, CA, Gooiker, GA, van Gijn, W et al: A systematic review and meta-analysis of the relationship between hospital/surgeon volume and outcome for radical cystectomy: an update for the ongoing debate. Eur Urol 2011, **59:** 775

70. Baazeem, A, Belzile, E, Ciampi, A et al: Varicocele and male factor infertility treatment: a new meta-analysis and review of the role of varicocele repair. Eur Urol 2011, **60:** 796

71. Abdel-Fattah, M, Ford, JA, Lim, CP et al: Single-incision mini-slings versus standard midurethral slings in surgical management of female stress urinary incontinence: a meta-analysis of effectiveness and complications. Eur Urol 2011, **60:** 468

72. Zhou, L, Wei, X, Sun, WJ et al: Selective Versus Hilar Clamping During Minimally Invasive Partial Nephrectomy: A Systematic Review and Meta-Analysis. J Endourol 2015, **29:** 855

73. Zhou, L, Cai, X, Li, H et al: Effects of alpha-Blockers, Antimuscarinics, or Combination Therapy in Relieving Ureteral Stent-Related Symptoms: A Meta-Analysis. J Endourol 2015, **29:** 650

74. Zhang, W, Zhou, T, Wu, T et al: Retrograde Intrarenal Surgery Versus Percutaneous Nephrolithotomy Versus Extracorporeal Shockwave Lithotripsy for Treatment of Lower Pole Renal Stones: A Meta-Analysis and Systematic Review. J Endourol 2015, **29:** 745

75. Lei, Y, Li, Z, Qi, L et al: The Prognostic Role of Ki-67/MIB-1 in Upper Urinary-Tract Urothelial Carcinomas: A Systematic Review and Meta-Analysis. J Endourol 2015, **29:** 1302

76. Chen, K, Mi, H, Xu, G et al: The Efficacy and Safety of Tamsulosin Combined with Extracorporeal Shockwave Lithotripsy for Urolithiasis: A Systematic Review and Meta-Analysis of Randomized Controlled Trials. J Endourol 2015, **29:** 1166

77. Zhang, K, Qi, E, Zhang, Y et al: Efficacy and safety of local steroids for urethra strictures: a systematic review and meta-analysis. J Endourol 2014, **28:** 962

78. Tang, Y, Li, J, Pu, C et al: Bipolar transurethral resection versus monopolar transurethral resection for benign prostatic hypertrophy: a systematic review and meta-analysis. J Endourol 2014, **28:** 1107

79. Tang, K, Xu, Z, Xia, D et al: Early outcomes of thulium laser versus transurethral resection of the prostate for managing benign prostatic hyperplasia: a systematic review and meta-analysis of comparative studies. J Endourol 2014, **28:** 65

80. Liu, W, Li, Y, Chen, M et al: Off-clamp versus complete hilar control partial nephrectomy for renal cell carcinoma: a systematic review and meta-analysis. J Endourol 2014, **28:** 567

81. Ishii, H, Rai, BP, Stolzenburg, JU et al: Robotic or open radical cystectomy, which is safer? A systematic review and meta-analysis of comparative studies. J Endourol 2014, **28:** 1215

82. Zhong, Q, Zheng, C, Mo, J et al: Total tubeless versus standard percutaneous nephrolithotomy: a meta-analysis. J Endourol 2013, **27:** 420

83. Yin, X, Tang, Z, Yu, B et al: Holmium: YAG laser lithotripsy versus pneumatic lithotripsy for treatment of distal ureteral calculi: a meta-analysis. J Endourol 2013, **27:** 408

84. Yin, L, Teng, J, Huang, CJ et al: Holmium laser enucleation of the prostate versus transurethral resection of the prostate: a systematic review and meta-analysis of randomized controlled trials. J Endourol 2013, **27:** 604

85. Wang, L, Wu, Z, Li, M et al: Laparoendoscopic single-site adrenalectomy versus conventional laparoscopic surgery: a systematic review and meta-analysis of observational studies. J Endourol 2013, **27:** 743

86. Hu, Q, Gou, Y, Sun, C et al: A systematic review and meta-analysis of current evidence comparing laparoendoscopic single-site adrenalectomy and conventional laparoscopic adrenalectomy. J Endourol 2013, **27:** 676

87. Aboumarzouk, OM, Hughes, O, Narahari, K et al: Safety and feasibility of laparoscopic radical cystectomy for the treatment of bladder cancer. J Endourol 2013, **27:** 1083

88. Zhang, X, Geng, J, Zheng, J et al: Photoselective vaporization versus transurethral resection of the prostate for benign prostatic hyperplasia: a meta-analysis. J Endourol 2012, **26:** 1109

89. Ding, H, Wang, Z, Du, W et al: NTrap in prevention of stone migration during ureteroscopic lithotripsy for proximal ureteral stones: a meta-analysis. J Endourol 2012, **26:** 130

90. Aboumarzouk, OM, Monga, M, Kata, SG et al: Flexible ureteroscopy and laser lithotripsy for stones >2 cm: a systematic review and meta-analysis. J Endourol 2012, **26:** 1257

91. Mei, H, Pu, J, Yang, C et al: Laparoscopic versus open pyeloplasty for ureteropelvic junction obstruction in children: a systematic review and meta-analysis. J Endourol 2011, **25:** 727

92. Luo, S, Lin, Y, Zhang, W: Does simultaneous transurethral resection of bladder tumor and prostate affect the recurrence of bladder tumor? A meta-analysis. J Endourol 2011, **25:** 291

93. Velazquez, N, Zapata, D, Wang, HH et al: Medical expulsive therapy for pediatric urolithiasis: Systematic review and meta-analysis. J Pediatr Urol 2015, **11:** 321

94. Desantis, DJ, Leonard, MP, Preston, MA et al: Effectiveness of biofeedback for dysfunctional elimination syndrome in pediatrics: a systematic review. J Pediatr Urol 2011, **7:** 342

95. Wang, HH, Gbadegesin, RA, Foreman, JW et al: Efficacy of antibiotic prophylaxis in children with vesicoureteral reflux: systematic review and meta-analysis. J Urol 2015, **193:** 963

96. van Osch, FH, Jochems, SH, van Schooten, FJ et al: Significant Role of Lifetime Cigarette Smoking in Worsening Bladder Cancer and Upper Tract Urothelial Carcinoma Prognosis: A Meta-Analysis. J Urol 2015

97. Seklehner, S, Laudano, MA, Xie, D et al: A meta-analysis of the performance of retropubic mid urethral slings versus transobturator mid urethral slings. J Urol 2015, **193:** 909

98. Massaro, PA, MacLellan, DL, Anderson, PA et al: Does intracytoplasmic sperm injection pose an increased risk of genitourinary congenital malformations in offspring compared to in vitro fertilization? A systematic review and meta-analysis. J Urol 2015, **193:** 1837

99. Fazeli, MS, Lin, Y, Nikoo, N et al: Biofeedback for nonneuropathic daytime voiding disorders in children: a systematic review and meta-analysis of randomized controlled trials. J Urol 2015, **193:** 274

100. de Bessa, J, Jr., de Carvalho Mrad, FC, Mendes, EF et al: Antibiotic prophylaxis for prevention of febrile urinary tract infections in children with vesicoureteral reflux: a meta-analysis of randomized, controlled trials comparing dilated to nondilated vesicoureteral reflux. J Urol 2015, **193:** 1772

101. Klatte, T, Shariat, SF, Remzi, M: Systematic review and meta-analysis of perioperative and oncologic outcomes of laparoscopic cryoablation versus laparoscopic partial nephrectomy for the treatment of small renal tumors. J Urol 2014, **191:** 1209

102. Hofmeester, I, Kollen, BJ, Steffens, MG et al: The association between nocturia and nocturnal polyuria in clinical and epidemiological studies: a systematic review and meta-analyses. J Urol 2014, **191:** 1028

103. Ebell, MH, Radke, T, Gardner, J: A systematic review of the efficacy and safety of desmopressin for nocturia in adults. J Urol 2014, **192:** 829

104. Zhu, S, Zhang, H, Xie, L et al: Risk factors and prevention of inguinal hernia after radical prostatectomy: a systematic review and meta-analysis. J Urol 2013, **189:** 884

105. Wang, X, Li, S, Liu, T et al: Laparoscopic pyelolithotomy compared to percutaneous nephrolithotomy as surgical management for large renal pelvic calculi: a meta-analysis. J Urol 2013, **190:** 888

106. Nguyen, T, Braga, LH, Hoogenes, J et al: Commercial video laparoscopic trainers versus less expensive, simple laparoscopic trainers: a systematic review and meta-analysis. J Urol 2013, **190:** 894

107. Morris, BJ, Wiswell, TE: Circumcision and lifetime risk of urinary tract infection: a systematic review and meta-analysis. J Urol 2013, **189:** 2118

108. Li, K, Lin, T, Zhang, C et al: Optimal frequency of shock wave lithotripsy in urolithiasis treatment: a systematic review and meta-analysis of randomized controlled trials. J Urol 2013, **190:** 1260

109. Filson, CP, Hollingsworth, JM, Clemens, JQ et al: The efficacy and safety of combined therapy with alpha-blockers and anticholinergics for men with benign prostatic hyperplasia: a meta-analysis. J Urol 2013, **190:** 2153

110. Beerepoot, MA, Geerlings, SE, van Haarst, EP et al: Nonantibiotic prophylaxis for recurrent urinary tract infections: a systematic review and meta-analysis of randomized controlled trials. J Urol 2013, **190:** 1981

111. Schauer, I, Madersbacher, S, Jost, R et al: The impact of varicocelectomy on sperm parameters: a meta-analysis. J Urol 2012, **187:** 1540

112. Sadeghi, R, Gholami, H, Zakavi, SR et al: Accuracy of sentinel lymph node biopsy for inguinal lymph node staging of penile squamous cell carcinoma: systematic review and meta-analysis of the literature. J Urol 2012, **187:** 25

113. Picozzi, S, Ricci, C, Gaeta, M et al: Upper urinary tract recurrence following radical cystectomy for bladder cancer: a meta-analysis on 13,185 patients. J Urol 2012, **188:** 2046

114. Mandava, SH, Serefoglu, EC, Freier, MT et al: Infection retardant coated inflatable penile prostheses decrease the incidence of infection: a systematic review and meta-analysis. J Urol 2012, **188:** 1855

115. Lu, Y, Tianyong, F, Ping, H et al: Antibiotic prophylaxis for shock wave lithotripsy in patients with sterile urine before treatment may be unnecessary: a systematic review and meta-analysis. J Urol 2012, **188:** 441

116. Kim, SP, Thompson, RH, Boorjian, SA et al: Comparative effectiveness for survival and renal function of partial and radical nephrectomy for localized renal tumors: a systematic review and meta-analysis. J Urol 2012, **188:** 51

117. Yakoubi, R, Lemdani, M, Monga, M et al: Is there a role for alpha-blockers in ureteral stent related symptoms? A systematic review and meta-analysis. J Urol 2011, **186:** 928

118. Shen, P, Jiang, M, Yang, J et al: Use of ureteral stent in extracorporeal shock wave lithotripsy for upper urinary calculi: a systematic review and meta-analysis. J Urol 2011, **186:** 1328

119. Pengfei, S, Yutao, L, Jie, Y et al: The results of ureteral stenting after ureteroscopic lithotripsy for ureteral calculi: a systematic review and meta-analysis. J Urol 2011, **186:** 1904

120. de Resende, JAJ, Cavalini, LT, Crispi, CP et al: Risk of urinary retention after nerve-sparing surgery for deep infiltrating endometriosis: A systematic review and meta-analysis. Neurourol Urodyn 2015

121. Cui, Y, Zhou, X, Zong, H et al: The efficacy and safety of onabotulinumtoxinA in treating idiopathic OAB: A systematic review and meta-analysis. Neurourol Urodyn 2015, **34:** 413

122. Burton, C, Sajja, A, Latthe, PM: Effectiveness of percutaneous posterior tibial nerve stimulation for overactive bladder: a systematic review and meta-analysis. Neurourol Urodyn 2012, **31:** 1206

123. Zhao, W, Yin, J, Yang, Z et al: Meta-analysis of Androgen Insensitivity in Preoperative Hormone Therapy in Hypospadias. Urology 2015, **85:** 1166

124. Yue, FG, Dong, L, Hu, TT et al: Efficacy of Dapoxetine for the treatment of premature ejaculation: a meta-analysis of randomized clinical trials on intravaginal ejaculatory latency time, patient-reported outcomes, and adverse events. Urology 2015, **85:** 856

125. Sun, Y, Luo, D, Yang, L et al: Efficacy of Phosphodiesterase-5 Inhibitor in Men With Premature Ejaculation: A New Systematic Review and Meta-analysis. Urology 2015, **86:** 947

126. Skolarikos, A, Grivas, N, Kallidonis, P et al: The Efficacy of Medical Expulsive Therapy (MET) in Improving Stone-free Rate and Stone Expulsion Time, After Extracorporeal Shock Wave Lithotripsy (SWL) for Upper Urinary Stones: A Systematic Review and Meta-analysis. Urology 2015, **86:** 1057

127. Scovell, JM, Mata, DA, Ramasamy, R et al: Association between the presence of sperm in the vasal fluid during vasectomy reversal and postoperative patency: a systematic review and meta-analysis. Urology 2015, **85:** 809

128. Herrel, LA, Goodman, M, Goldstein, M et al: Outcomes of microsurgical vasovasostomy for vasectomy reversal: a meta-analysis and systematic review. Urology 2015, **85:** 819

129. Cui, X, Ji, F, Yan, H et al: Comparison between extracorporeal shock wave lithotripsy and ureteroscopic lithotripsy for treating large proximal ureteral stones: a meta-analysis. Urology 2015, **85:** 748

130. Cao, D, Liu, L, Hu, Y et al: A systematic review and meta-analysis of circumcision with Shang Ring vs conventional circumcision. Urology 2015, **85:** 799

131. Bai, Y, Pu, C, Yuan, H et al: Assessing the Impact of Barbed Suture on Vesicourethral Anastomosis During Minimally Invasive Radical Prostatectomy: A Systematic Review and Meta-analysis. Urology 2015, **85:** 1368

132. Bai, Y, Pu, C, Han, P et al: Selective Serotonin Reuptake Inhibitors Plus Phosphodiesterase-5 Inhibitors for Premature Ejaculation: A Systematic Review and Meta-analysis. Urology 2015, **86:** 758

133. Wu, XJ, Zhi, Y, Zheng, J et al: Dutasteride on benign prostatic hyperplasia: a meta-analysis on randomized clinical trials in 6460 patients. Urology 2014, **83:** 539

134. Hao, N, Tian, Y, Liu, W et al: Antimuscarinics and alpha-blockers or alpha-blockers monotherapy on lower urinary tract symptoms--a meta-analysis. Urology 2014, **83:** 556

135. Barrett, K, Braga, LH, Farrokhyar, F et al: Primary realignment vs suprapubic cystostomy for the management of pelvic fracture-associated urethral injuries: a systematic review and meta-analysis. Urology 2014, **83:** 924

136. Xin, Z, Huang, Y, Lu, J et al: Addition of antimuscarinics to alpha-blockers for treatment of lower urinary tract symptoms in men: a meta-analysis. Urology 2013, **82:** 270

137. Tsai, HT, Penson, DF, Makambi, KH et al: Efficacy of intermittent androgen deprivation therapy vs conventional continuous androgen deprivation therapy for advanced prostate cancer: a meta-analysis. Urology 2013, **82:** 327

138. Pu, C, Yang, L, Liu, L et al: Topical anesthetic agents for premature ejaculation: a systematic review and meta-analysis. Urology 2013, **81:** 799

139. Zhu, YP, Yao, XD, Zhang, SL et al: Pelvic floor electrical stimulation for postprostatectomy urinary incontinence: a meta-analysis. Urology 2012, **79:** 552

140. Wu, T, Yue, X, Duan, X et al: Efficacy and safety of tramadol for premature ejaculation: a systematic review and meta-analysis. Urology 2012, **80:** 618

141. Li, F, Chiba, K, Yamaguchi, K et al: Effect of varicocelectomy on testicular volume in children and adolescents: a meta-analysis. Urology 2012, **79:** 1340

142. Ding, H, Du, W, Wang, H et al: Efficacy and safety of udenafil for erectile dysfunction: a meta-analysis of randomized controlled trials. Urology 2012, **80:** 134

143. Tang, L, Gao, X, Xu, B et al: Placement of ureteral stent after uncomplicated ureteroscopy: do we really need it? Urology 2011, **78:** 1248

144. Ni, S, Qiyin, C, Tao, W et al: Tubeless percutaneous nephrolithotomy is associated with less pain and shorter hospitalization compared with standard or small bore drainage: a meta-analysis of randomized, controlled trials. Urology 2011, **77:** 1293

145. Liu, L, Zheng, S, Han, P et al: Phosphodiesterase-5 inhibitors for lower urinary tract symptoms secondary to benign prostatic hyperplasia: a systematic review and meta-analysis. Urology 2011, **77:** 123

146. Li, F, Li, C, Jiang, Z et al: XRCC3 T241M polymorphism and bladder cancer risk: a meta-analysis. Urology 2011, **77:** 511.e1

147. Li, F, An, SL, Zhou, Y et al: Milk and dairy consumption and risk of bladder cancer: a meta-analysis. Urology 2011, **78:** 1298

148. Zhu, W, Liu, Y, Liu, L et al: Minimally invasive versus standard percutaneous nephrolithotomy: a meta-analysis. Urolithiasis 2015, **43:** 563

149. Zheng, C, Yang, H, Luo, J et al: Extracorporeal shock wave lithotripsy versus retrograde intrarenal surgery for treatment for renal stones 1-2 cm: a meta-analysis. Urolithiasis 2015, **43:** 549

150. Pu, C, Wang, J, Tang, Y et al: The efficacy and safety of percutaneous nephrolithotomy under general versus regional anesthesia: a systematic review and meta-analysis. Urolithiasis 2015, **43:** 455

151. Mi, Y, Ren, K, Pan, H et al: Flexible ureterorenoscopy (F-URS) with holmium laser versus extracorporeal shock wave lithotripsy (ESWL) for treatment of renal stone <2 cm: a meta-analysis. Urolithiasis 2015

152. Lu, P, Wang, Z, Song, R et al: The clinical efficacy of extracorporeal shock wave lithotripsy in pediatric urolithiasis: a systematic review and meta-analysis. Urolithiasis 2015, **43:** 199

153. Liu, LH, Kang, R, He, J et al: Diabetes mellitus and the risk of urolithiasis: a meta-analysis of observational studies. Urolithiasis 2015, **43:** 293

154. Deng, T, Liao, B, Tian, Y et al: New-onset diabetes mellitus after shock wave lithotripsy for urinary stone: a systematic review and meta-analysis. Urolithiasis 2015, **43:** 227

155. Zhang, X, Xia, L, Xu, T et al: Is the supine position superior to the prone position for percutaneous nephrolithotomy (PCNL)? Urolithiasis 2014, **42:** 87

156. Yu, C, Xu, Z, Long, W et al: Hemostatic agents used for nephrostomy tract closure after tubeless PCNL: a systematic review and meta-analysis. Urolithiasis 2014, **42:** 445

157. Wang, J, Zhang, C, Tan, G et al: The use of adjunctive hemostatic agents in tubeless percutaneous nephrolithotomy: a meta-analysis. Urolithiasis 2014, **42:** 509

158. Liu, W, Chen, M, Li, M et al: Vitamin D receptor gene (VDR) polymorphisms and the urolithiasis risk: an updated meta-analysis based on 20 case-control studies. Urolithiasis 2014, **42:** 45

159. Li, Y, Yang, L, Xu, P et al: One-shot versus gradual dilation technique for tract creation in percutaneous nephrolithotomy: a systematic review and meta-analysis. Urolithiasis 2013, **41:** 443

160. Dehong, C, Liangren, L, Huawei, L et al: A comparison among four tract dilation methods of percutaneous nephrolithotomy: a systematic review and meta-analysis. Urolithiasis 2013, **41:** 523

161. Chua, ME, Park, JH, Castillo, JC et al: Terpene compound drug as medical expulsive therapy for ureterolithiasis: a meta-analysis. Urolithiasis 2013, **41:** 143

162. Song, T, Liao, B, Zheng, S et al: Meta-analysis of postoperatively stenting or not in patients underwent ureteroscopic lithotripsy. Urol Res 2012, **40:** 67

163. Picozzi, SC, Ricci, C, Gaeta, M et al: Urgent shock wave lithotripsy as first-line treatment for ureteral stones: a meta-analysis of 570 patients. Urol Res 2012, **40:** 725

164. Picozzi, SC, Ricci, C, Gaeta, M et al: Urgent ureteroscopy as first-line treatment for ureteral stones: a meta-analysis of 681 patients. Urol Res 2012, **40:** 581

165. Yuan, H, Zheng, S, Liu, L et al: The efficacy and safety of tubeless percutaneous nephrolithotomy: a systematic review and meta-analysis. Urol Res 2011, **39:** 401

166. Yang, L, Zhu, Y, Tang, Z et al: Antibiotics may not decrease prostate-specific antigen levels or prevent unnecessary prostate biopsy in patients with moderately increased prostate-specific antigen levels: A meta-analysis. Urol Oncol 2015, **33:** 201.e17

167. Yuan, H, Chen, X, Liu, L et al: Risk factors for intravesical recurrence after radical nephroureterectomy for upper tract urothelial carcinoma: a meta-analysis. Urol Oncol 2014, **32:** 989

168. Mandel, P, Tilki, D, Eslick, GD: Extent of lymph node dissection and recurrence-free survival after radical cystectomy: a meta-analysis. Urol Oncol 2014, **32:** 1184

169. Kim, HS, Kim, M, Jeong, CW et al: Presence of lymphovascular invasion in urothelial bladder cancer specimens after transurethral resections correlates with risk of upstaging and survival: a systematic review and meta-analysis. Urol Oncol 2014, **32:** 1191

170. Hu, Q, Gou, Y, Sun, C et al: The prognostic value of C-reactive protein in renal cell carcinoma: a systematic review and meta-analysis. Urol Oncol 2014, **32:** 50.e1

171. Song, T, Yin, Y, Liao, B et al: Capsular invasion in renal cell carcinoma: a meta-analysis. Urol Oncol 2013, **31:** 1321

172. Zhu, Y, Zhuo, J, Xu, D et al: Thulium laser versus standard transurethral resection of the prostate for benign prostatic obstruction: a systematic review and meta-analysis. World J Urol 2015, **33:** 509

173. Picozzi, SC, Ricci, C, Bonavina, L et al: Feasibility and outcomes regarding open and laparoscopic radical prostatectomy in patients with previous synthetic mesh inguinal hernia repair: meta-analysis and systematic review of 7,497 patients. World J Urol 2015, **33:** 59

174. Lucca, I, Shariat, SF, Hofbauer, SL et al: Outcomes of minimally invasive simple prostatectomy for benign prostatic hyperplasia: a systematic review and meta-analysis. World J Urol 2015, **33:** 563

175. Lin, Y, Wu, X, Xu, A et al: Transurethral enucleation of the prostate versus transvesical open prostatectomy for large benign prostatic hyperplasia: a systematic review and meta-analysis of randomized controlled trials. World J Urol 2015

176. Li, HJ, Kang, DY: Prevalence of sexual dysfunction in men with chronic prostatitis/chronic pelvic pain syndrome: a meta-analysis. World J Urol 2015

177. Chang, SJ, Hsu, CK, Hsieh, CH et al: Comparing the efficacy and safety between robotic-assisted versus open pyeloplasty in children: a systemic review and meta-analysis. World J Urol 2015, **33:** 1855

178. Carneiro, A, Sasse, AD, Wagner, AA et al: Cardiovascular events associated with androgen deprivation therapy in patients with prostate cancer: a systematic review and meta-analysis. World J Urol 2015, **33:** 1281

179. Bouwman, II, Voskamp, MJ, Kollen, BJ et al: Do lower urinary tract symptoms predict cardiovascular diseases in older men? A systematic review and meta-analysis. World J Urol 2015, **33:** 1911

180. Park, T, Choi, JY: Efficacy and safety of dutasteride for the treatment of symptomatic benign prostatic hyperplasia (BPH): a systematic review and meta-analysis. World J Urol 2014, **32:** 1093

181. Picozzi, SC, Ricci, C, Stubinski, R et al: Is stone diameter a variable in the decision process of employing a ureteral stent in patients undergoing uncomplicated ureterorenoscopy and associated intracorporeal lithotripsy? World J Urol 2013, **31:** 1617

182. Novara, G, Tubaro, A, Sanseverino, R et al: Systematic review and meta-analysis of randomized controlled trials evaluating silodosin in the treatment of non-neurogenic male lower urinary tract symptoms suggestive of benign prostatic enlargement. World J Urol 2013, **31:** 997

183. Liu, B, Mao, Q, Lin, Y et al: The association of cruciferous vegetables intake and risk of bladder cancer: a meta-analysis. World J Urol 2013, **31:** 127
